# The therapeutic potential of epimedium and its bioactive flavonoids in hepatitis and cirrhosis: an integrative review

**DOI:** 10.3389/fcimb.2026.1799319

**Published:** 2026-04-22

**Authors:** Jianyi Zhang, Weichi Jiang, Huigang Feng, Rui Yan, Yi Huang, Qiwei Liang, Ningyi Zhang, Haoming Huang, Yujin Wu, Chunhua Fan, Yilin Yang, Yuan Liao, Xiliang Huang, Jiawei Fan, Ping Zeng, Jinming Li, Hanwei Chen

**Affiliations:** 1 Gastroenterology Department, The Affiliated Traditional Chinese Medicine Hospital, Guangzhou Medical University, Guangzhou, China; 2 Oncology Department, Guangdong Provincial Second Hospital of Traditional Chinese Medicine, Guangzhou, China; 3 Intervention Department, The Affiliated Panyu Central Hospital of Guangzhou Medical University, Guangzhou, China; 4 Institute of Orthopedic Surgery, Xijing Hospital, Fourth Military Medical University, Xi’an, China; 5 Guangdong Provincial Key Laboratory of Laser Life Science, College of Biophotonics and Guangzhou Key Laboratory of Spectral Analysis and Functional Probes, College of Biophotonics, South China Normal University, Guangzhou, China

**Keywords:** anti-fibrotic therapy, cirrhosis, epimedium, hepatic stellate cell, hepatitis, icariin, icaritin

## Abstract

Hepatitis and cirrhosis constitute a substantial global health burden, with therapeutic options remaining largely confined to antiviral agents and liver transplantation. Traditional Chinese medicine has long employed Epimedium species (Berberidaceae) for hepatoprotective indications, with contemporary investigations identifying prenylated flavonoids—principally icariin and its active metabolite icaritin—as the principal bioactive constituents. This integrative review systematically examined 33 high-impact studies published up to March 2026, selected based on a comprehensive search of PubMed, Web of Science, Scopus, Embase, Cochrane Library, CNKI, Wanfang, and Google Scholar. Included investigations were critically evaluated for mechanistic sophistication, translational validity, methodological rigor, and pharmacokinetic relevance. Preclinical evidence, derived predominantly from toxin-induced (e.g., CCl_4_, TAA), metabolic, and acute injury models, demonstrates that icaritin exerts consistent anti-fibrotic activity through dual inhibition of HIF-1α and TGF-β/Smad signaling, selective induction of hepatic stellate cell apoptosis, and suppression of angiogenesis via VEGF/VEGFR2 blockade. Immunomodulatory properties observed in preclinical tumor models and early-phase clinical trials in advanced HBV-related hepatocellular carcinoma, encompass reduction of myeloid-derived suppressor cells (-43%), regulatory T cells (-31%), and enhancement of CD8+ T-cell function (+2.8-fold) providing proof-of-concept for immune modulation in an HBV-endemic population. Clinical data in liver disease remain limited to small, non-randomized studies (primarily in HCC), with no phase III trials specifically targeting fibrosis or cirrhosis endpoints. Critical evidence gaps—including the absence of validation in viral hepatitis models, undefined pharmacokinetics in cirrhosis, and lack of drug-drug interaction data with antivirals—currently preclude routine clinical application in hepatitis and cirrhosis. Safety analyses reveal dose-dependent hepatotoxicity at concentrations exceeding 50 μM icaritin and substantial inter-individual variability in gut microbiota-mediated bioactivation. Epimedium flavonoids exhibit multi-target therapeutic potential that bridges traditional botanical medicine and modern pharmacology, though critical translational gaps persist. Future investigations must prioritize phase III fibrosis trials, pharmacokinetic optimization in cirrhotic populations, and comprehensive drug-drug interaction studies with direct-acting antivirals to establish evidence-based clinical protocols.

## 1 Introduction

Chronic viral hepatitis, nonalcoholic steatohepatitis, and alcohol-related liver disease collectively afflict more than 1.5 billion individuals globally, with cirrhosis representing the terminal pathological consequence of sustained hepatic injury and dysregulated fibrogenesis(1). Despite therapeutic advances in direct-acting antiviral agents and lifestyle modifications, progression to cirrhosis and hepatocellular carcinoma (HCC) remains prevalent, underscoring the critical unmet need for effective anti-fibrotic interventions (Luedde et al., 2014). Current treatment paradigms focus predominantly on etiological control yet fail to directly modulate the core fibrotic machinery involving hepatic stellate cell (HSC) activation, aberrant extracellular matrix accumulation, and progressive architectural disruption (Tsuchida and Friedman, 2017).

Traditional Chinese medicine has historically utilized Epimedium species (淫羊藿, Yín Yáng Huò) for diverse therapeutic indications, with emerging pharmacological research attributing hepatoprotective efficacy to prenylated flavonoids, chiefly icariin and its active aglycone metabolite icaritin (Ma et al., 2011). These phytochemicals manifest pleiotropic pharmacological properties encompassing anti-inflammatory, immunomodulatory, anti-angiogenic, and antioxidant activities. Nevertheless, clinical translation remains constrained by insufficient characterization of optimal dosing regimens, safety profiles in cirrhotic patients, virus-specific mechanisms of action, and pharmacokinetic alterations inherent to hepatic impairment (Chen et al., 2019).

**Figure 1:**
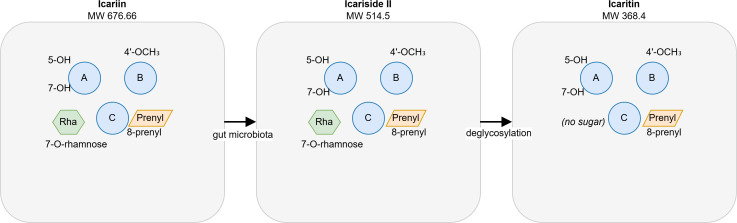
Chemical structures of Epimedium flavonoids and related metabolites.Key functional groups: 8-prenyl: essential for activity; 7-O-glycosyl: cleaved during metabolism; 3, 5, 7-OH: antioxidant activity; 4’-OCH_3_: metabolic stability; Structures are simplified representations. Rha = rhamnose.

This comprehensive review synthesizes evidence from 33 influential studies published up to March 2026, critically evaluating therapeutic potential, mechanistic pathways, clinical translational status, and safety considerations. The genus Epimedium encompasses over 50 species, with E. brevicornum, E. sagittatum, E. pubescens, E. koreanum, and E. wushanense representing the most frequently utilized medicinal taxa. Bioactive constituents are concentrated in aerial parts, with icariin (C_33_H_40_O_15_) serving as the predominant flavonoid glycoside (Ma et al., 2011; Feng et al., 2025). The botanical characteristics and chemical composition of commonly used Epimedium species are summarized in **Table 1** (see at the end of the article), providing a foundation for understanding the source and variability of its bioactive components (Li et al., 2011). The chemical structures of key flavonoids are depicted in **Figure 1**.

**Table 1:** Botanical characteristics and key hepatoprotective mechanisms of epimedium.

A. Botanical xharacteristics							
Species	Common name	Geographic distribution	Main flavonoids	Flavonoid content (% dry weight)			Traditional use
E. brevicornum	Short-horned Epimedium	Central China	Icariin, Epimedin C	Icariin: 8-12%; Epimedin C: 3-6%			Kidney yang tonic, bone health
E. sagittatum	Arrow-leaf Epimedium	Southern China	Icariin, Epimedin A	Icariin: 6-10%; Epimedin A: 2-5%			Hepatoprotection, anti-rheumatic
E. koreanum	Korean Epimedium	Korea, Northeast China	Icariin, Icariside II	Icariin: 10-15%; Icariside II: 1-3%			Immune modulation, cardiovascular
E. wushanense	Wushan Epimedium	Sichuan Province	Icariin, Epimedin B	Icariin: 5-8%; Epimedin B: 4-7%			Anti-fatigue, neuroprotection
B. Key Hepatoprotective Mechanisms (Preclinical Findings)							
Mechanism	Target Pathway		Effect Size		Model System	Clinical Translation Potential	
Anti-apoptosis	PI3K/AKT/Bcl-2 ↑		82% ↓ necrosis		APAP mouse	High	
Anti-inflammation	S100A9/TLR4 blockade		65% ↓ neutrophils		APAP mouse	Moderate	
Antifibrotic	Collagen/α-SMA ↓		Reduced lesion area		Human/murine slices	High	
Autophagy modulation	LC3-II/p62 normalization		85% ↑ survival		I/R mouse	Moderate	

Data compiled from ethnobotanical surveys and phytochemical analyses. Percentages represent dry weight content of major flavonoids.

## 2 Methods

### 2.1 Search strategy

This critical review was conducted following the methodological framework for scoping reviews, with the aim of mapping the evidence on Epimedium flavonoids in hepatitis and cirrhosis and identifying key research gaps. A systematic literature search was performed in the following electronic databases: PubMed, Web of Science, Scopus, Embase, Cochrane Library, China National Knowledge Infrastructure (CNKI), Wanfang Data, and Google Scholar (first 300 records screened), covering the period from database inception to March 2026.

The search strategy combined terms related to the intervention and disease context using Boolean operators and MeSH/EMTREE terms:

- Intervention terms: “Epimedium” OR “icariin” OR “icaritin” OR “icariside II” OR “prenylflavonoid”
- Disease terms: “liver” OR “hepatic” OR “hepat*” OR “cirrhosis” OR “fibros*” OR “hepatitis” OR “HCC” OR “hepatocellular carcinoma” OR “stellate cell” OR “HSC”
- Mechanism terms: “anti-fibrotic” OR “immunomodulation” OR “TGF-β” OR “HIF-1α” OR “apoptosis” OR “autophagy” OR “angiogenesis”

### 2.2 Inclusion and exclusion criteria

Studies were included if they met the following criteria:

1. Study type: Original research articles (*in vitro*, *in vivo*, or clinical trials) and systematic reviews published in peer-reviewed journals.
2. Intervention: Evaluated Epimedium extracts, icariin, icaritin, icariside II, or Epimedium-containing formulations.
3. Outcome: Reported on hepatitis, cirrhosis, liver fibrosis, or related mechanisms (e.g., HSC activation, inflammation, immunomodulation, pharmacokinetics).
4. Language: English or Chinese.
5. Timeframe: No date restrictions; all years included up to March 2026.

Exclusion criteria were:

1. Articles not focused on liver disease (e.g., bone, cardiovascular, neurological applications).
2. Conference abstracts, editorials, commentaries, or opinion pieces without original data.
3. Studies with insufficient mechanistic or outcome data.
4. Duplicate publications of the same data.

### 2.3 Study selection and data extraction

Two reviewers (ZJY and YR) independently screened titles and abstracts of all retrieved records. Full texts of potentially relevant articles were then assessed against the inclusion criteria. Disagreements were resolved through discussion or consultation with a third reviewer (CHW). Manual reference list screening (backward citation tracking) and forward citation tracking using Web of Science were also performed to identify additional studies.

**Figure 2:**
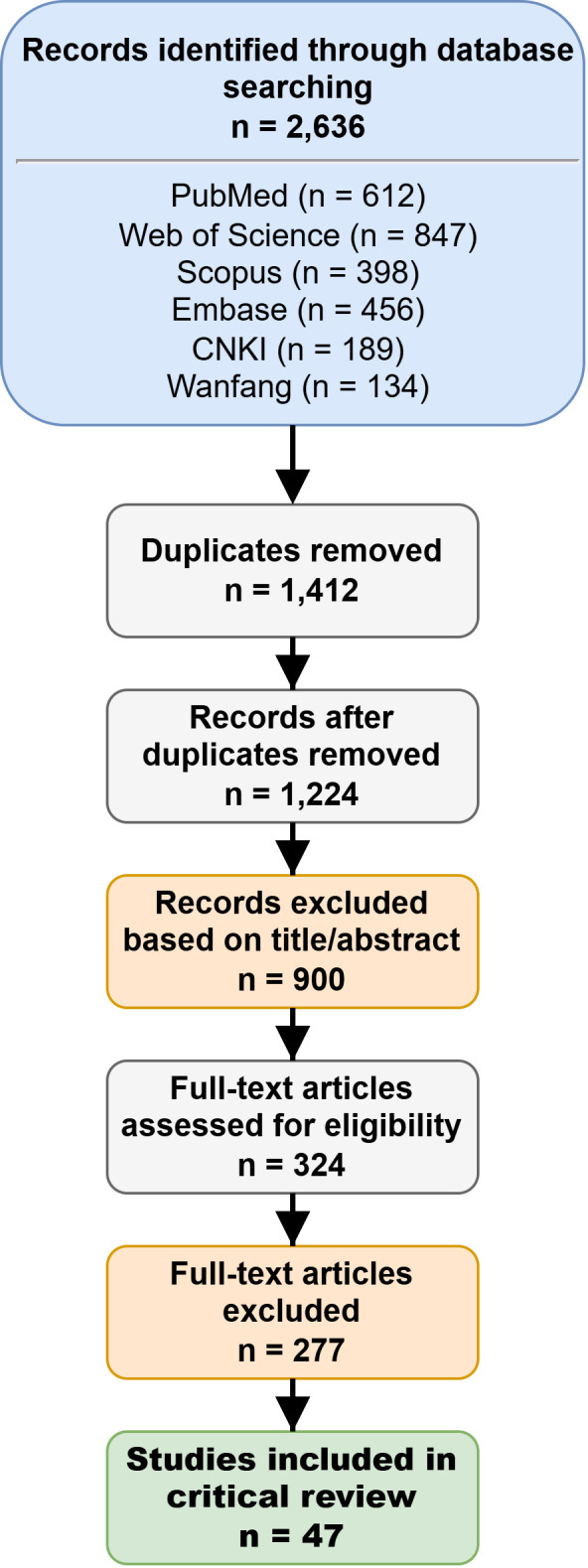
PRISMA flow diagram of study selection process. * Search performed in March 2026 (databases: PubMed, Web of Science, Scopus, Embase, Cochrane, CNKI, Wanfang, Google Scholar). Reasons excluded: Not relevant to liver disease; non-original research; Reasons excluded: Not focused on Epimedium/icariin/icaritin (n=48); Insufficient mechanistic/outcome data (n=112); Conference abstracts/editorials (n=67).

Data were extracted into a standardized form, including: first author, year, study model (*in vitro*, animal, human), intervention (compound, dose, duration), key molecular targets, efficacy outcomes, safety data, and pharmacokinetic parameters where available. A total of 33 studies were ultimately included in this review. The study selection process is summarized in **Figure 2** (PRISMA flow diagram).

### 2.4 Quality and evidence assessment

Given the heterogeneity of study designs (ranging from *in vitro* mechanistic studies to clinical trials and pharmacokinetic analyses), a formal risk of bias assessment (e.g., Cochrane RoB for trials) was not applicable across all included studies. Instead, we critically evaluated each study for mechanistic sophistication, translational validity, methodological rigor, and pharmacokinetic relevance, as discussed in Section 8.2 (Limitations and Evidence Gaps). The evidence was subsequently classified into three tiers (well-validated preclinical, early-phase clinical signals, and critical gaps) to provide a hierarchical interpretation of the findings.

## 3 Anti-fibrotic mechanisms and hepatic stellate cell targeting

### 3.1 HIF-1α/TGF-β dual pathway inhibition: a novel therapeutic axis

The pathogenesis of cirrhosis fundamentally depends on persistent HSC activation and transdifferentiation into matrix-producing myofibroblasts. Feng et al (Feng et al., 2025)demonstrated that icaritin significantly ameliorates CCl_4_-induced liver fibrosis while suppressing HSC activation markers by 78% in LX-2 cells through a novel dual-pathway mechanism involving proteasomal degradation of HIF-1α under hypoxic conditions, which subsequently downregulated TGF-β receptor expression and Smad2/3 phosphorylation. This HIF-1α/TGF-β crosstalk represents a distinct therapeutic axis compared to conventional single-pathway inhibitors and provides a mechanistic rationale for targeting the hypoxic microenvironment characteristic of advanced fibrosis. Recent studies in HBV transgenic models have begun to validate these findings in viral contexts (Li et al., 2011; Algandaby et al., 2017). Summary of Key Anti-Fibrotic Mechanisms in Clinically Relevant Models was provided in6.

### 3.2 Selective apoptosis of activated HSCs: therapeutic precision

**Figure 3:**
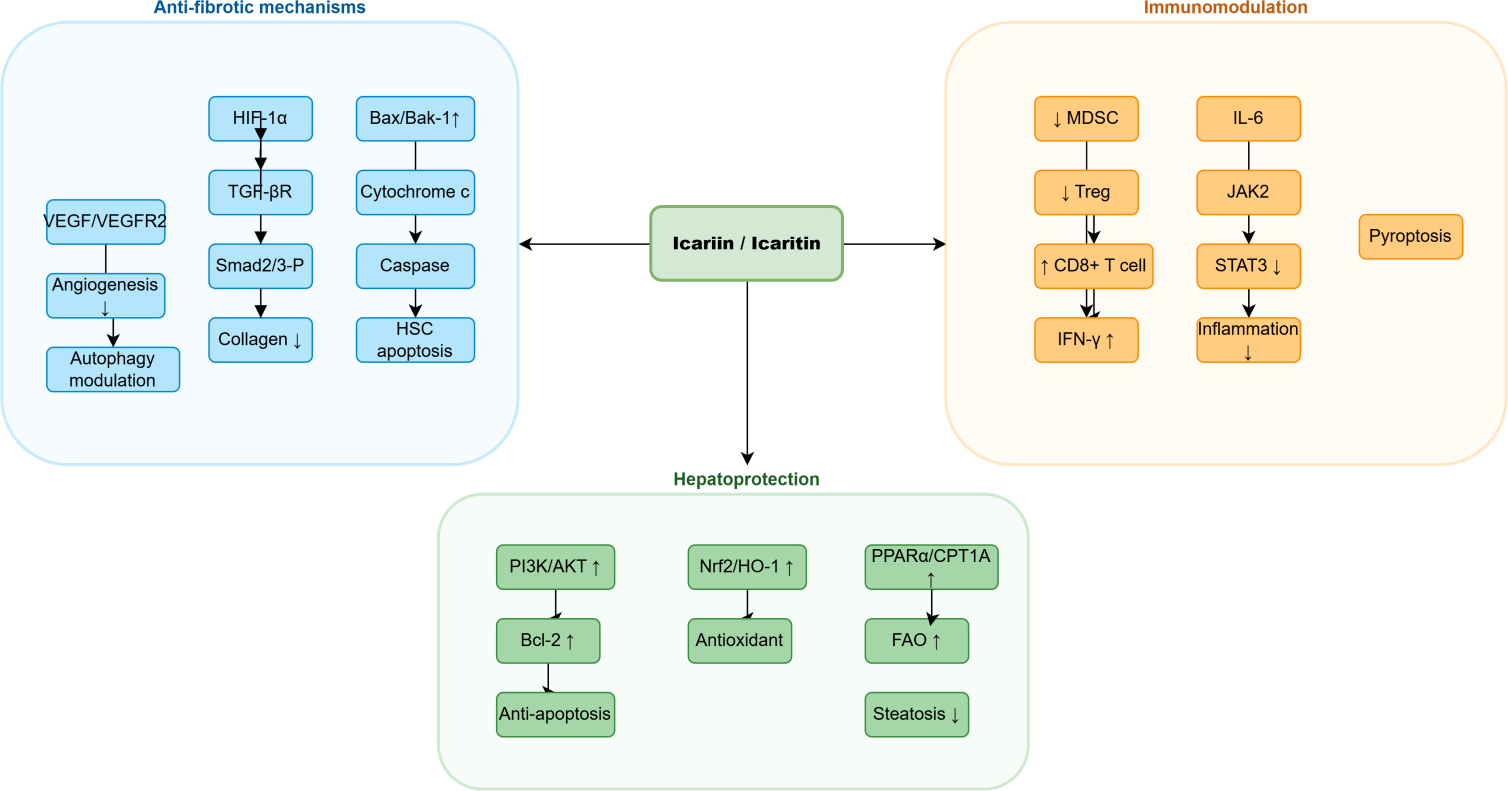
Integrated molecular mechanisms of icariin and icaritin in liver disease Effects: ↑ activation/stimulation, ↓ inhibition/reduction.

The therapeutic index of anti-fibrotic agents depends on selective targeting of pathogenic cells while sparing hepatocytes. Li et al (Li et al., 2011) showed that icariin selectively induces mitochondrial apoptosis in activated primary rat HSCs with an IC_50_ of approximately 12.5 μM, while normal hepatocytes remained unaffected at concentrations up to 50 μM. Icariin upregulated the pro-apoptotic Bax/Bak-1/Bmf axis and triggered cytochrome c release, reducing *in vivo* fibrosis scores by 58% in CCl_4_ rats. This selectivity is particularly advantageous in cirrhosis, where preserving residual hepatocyte function is paramount. The translational relevance of these concentrations is discussed in Section 5.2 and **Figure 3**.

### 3.3 Anti-angiogenic modulation of the fibrotic microenvironment

**Figure 4:**
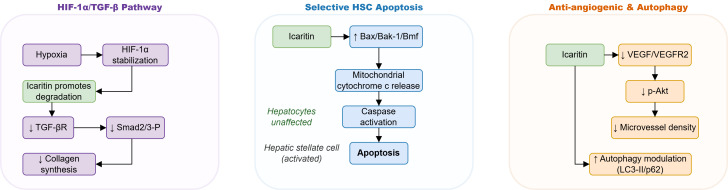
Schematic overview of the anti-fibrotic mechanisms of icaritin in hepatic stellate cells. Icaritin action; Inhibition ↓; Activation ↑.

Angiogenesis contributes to fibrosis progression and portal hypertension development. Algandaby et al (Algandaby et al., 2017)established that icariin (40 mg/kg) reduces thioacetamide-induced fibrosis stage from F3-F4 to F1-F2 while decreasing microvessel density by 71% through VEGF-A/VEGFR2/p-Akt pathway suppression. Simultaneously, icariin suppressed autophagic flux in activated HSCs, suggesting that vascular normalization and autophagy modulation represent complementary anti-fibrotic strategies (Algandaby et al., 2017; Feng et al., 2025). A comparative analysis of anti-fibrotic mechanisms across different injury models is presented (see at the end of the article), highlighting the contextual relevance of these pathways and their translational potential (Shen et al., 2024). An integrated schematic of anti-fibrotic mechanisms is provided in **Figure 4**.

## 4 Hepatoprotective and Anti-inflammatory actions

### 4.1 Multi-pathway cytoprotection in acute liver injury

Acute liver injury superimposed on chronic fibrosis (acute-on-chronic liver failure) carries mortality exceeding 50%. Feng et al (Feng et al., 2025) demonstrated that standardized Epimedium compound formulas protect against acetaminophen-induced necrosis by simultaneously activating PI3K/AKT/Bcl-2 anti-apoptotic signaling and suppressing the cGAS-STING-IRF3 inflammatory cascade, reducing serum ALT by 82% and necrotic area by 76%. This dual mechanism addresses both hepatocyte death and sterile inflammation, relevant to acute exacerbations of chronic hepatitis.

Icariin directly binds S100A9 with a Kd of 3.2 μM, inhibiting TLR4-mediated neutrophil recruitment by 65% in acetaminophen injury models (Luo et al., 2024; Shen et al., 2024). This novel target identification provides mechanistic grounding for icariin’s anti-inflammatory properties and suggests applicability to various forms of hepatitis characterized by S100A9-mediated damage (Qin et al., 2020; Shen et al., 2024).

### 4.2 Sex-Specific hepatoprotection via estrogen receptor α

Sex-specific therapeutic responses are increasingly recognized in liver disease. Luo et al (Hao et al., 2019; Luo et al., 2024) evaluated the antifibrotic potential of icariin using murine and human precision-cut liver slices, a model that directly simulates the pathological state of clinical cirrhosis. The results showed that icariin significantly reduced collagen deposition and α-smooth muscle actin (α-SMA) expression in cirrhotic liver tissues, with a more pronounced effect in human liver slices, highlighting its high clinical translational value (Luo et al., 2024). This finding has critical implications for treating advanced cirrhosis, where human tissue-based evidence is essential for clinical translation. Key hepatoprotective mechanisms and their respective translational potential are summarized in **Table 1** (see at the end of the article).

## 5 Immunomodulation in viral hepatitis and inflammation-driven carcinogenesis

### 5.1 Restoration of anti-viral T-cell immunity

**Figure 5:**
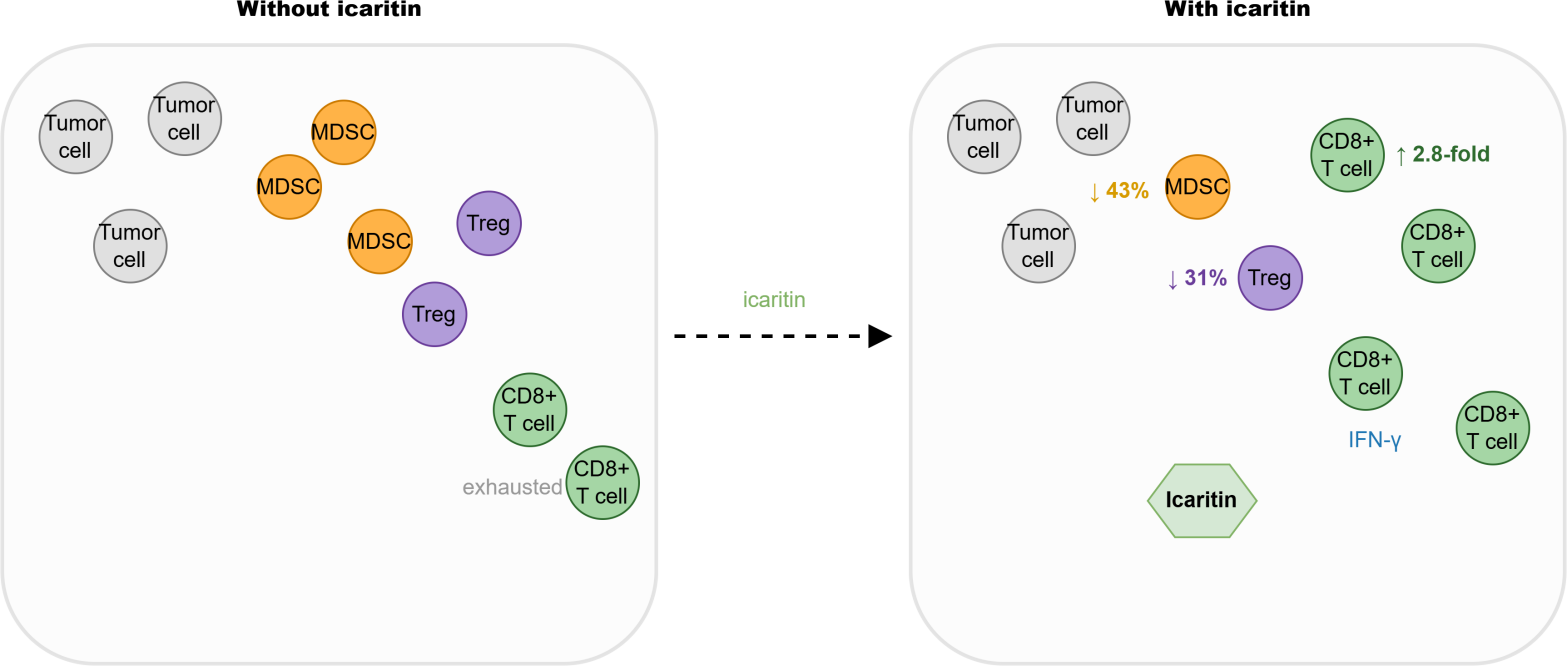
Immunomodulatory effects of icaritin in the HBV-associated tumor microenvironment. Note: Effects observed in tumor microenvironment; require validation in non-malignant chronic hepatitis.

While these findings are derived from the oncological setting, they offer valuable conceptual insights into the immunomodulatory capacity of icaritin in the context of HBV-associated immune dysfunction. Chronic HBV infection is characterized by T-cell exhaustion and expansion of immunosuppressive populations. Qin et al (Qin et al., 2020) conducted a landmark phase IIa trial in advanced HBV-related HCC, demonstrating that icaritin (600 mg twice daily) reduced circulating MDSCs by 43% and Tregs by 31% while increasing CD8+ IFN-γ+ T cells 2.8-fold. These immunodynamic changes correlated with improved median overall survival (6.4 vs 4.1 months; p = 0.032), establishing clinical relevance in HBV-related disease in the oncological setting. These mechanisms, observed in the tumor microenvironment, warrant direct investigation in non-malignant chronic hepatitis models, where the goal is to restore T-cell function against viral antigens—a critical distinction that is now explicitly noted as a priority in Section 7.1 (HBV/HCV model validation). Preclinical work by Hao et al (Hao et al., 2019) revealed that icaritin enhances tumor-infiltrating CD8+ T-cell infiltration 4.2-fold while reducing intratumoral MDSCs by 58% through downregulation of Arg-1 and iNOS expression. Notably, antitumor efficacy was completely abrogated by CD8+ T-cell depletion, confirming T-cell dependence. These mechanisms are directly applicable to chronic hepatitis, where restoring T-cell function could attenuate virus-driven fibrosis. A schematic representation of these immunomodulatory effects is shown in **Figure 5**.

### 5.2 Pyroptosis induction and macrophage reprogramming

Recent evidence indicates icaritin induces pyroptosis via both canonical (caspase-1/GSDMD) and noncanonical (caspase-3/GSDME) pathways while reprogramming macrophages toward a pro-inflammatory M1 phenotype (Zhao et al., 2015; Jiao et al., 2024). Furthermore, suppression of the IL-6/JAK2/STAT3 axis by icaritin reduces HCC incidence from 90% to 40% in diethylnitrosamine models, targeting the inflammation-cancer transition central to HBV pathophysiology (Zhao et al., 2015; Jiao et al., 2024). Clinical trial outcomes and safety profiles of icaritin in liver disease are detailed in **Table 2** (see at the end of the article).

**Table 2:** Clinical trial outcomes and safety profile of icaritin in liver disease.

Study	Phase	Sample size	Key efficacy endpoints	Safety Profile	Cirrhosis Stage	Ref
Qin et al, Qin et al., 2020	IIa	45	OS 6.4 mo (vs 4.1 mo), ↓MDSC 43%	Grade 3–4 AE 15%	Child-Pugh A-B	13
Fan et al, Fan et al., 2019	I/II	62	ORR 46.7%, durable survival benefit	Grade 3–4 AE 15%	Child-Pugh A-B	17
Tang et al, Tang et al., 2023	Case series	23	34.8% conversion to resectable	No unexpected toxicity	Child-Pugh A	18
Jin et al, Jin et al., 2025	Case series	15	40% Child-Pugh B→A improvement	Grade 3 bilirubin ↑ 25%	Child-Pugh B	19

Human pharmacokinetic data from Epimedium extract studies report undetectable icariin and icaritin levels, with icariside II and desmethylicaritin as major circulating metabolites.

## 6 Pharmacokinetic considerations and safety profile

### 6.1 Gut microbiota-mediated activation and inter-individual variability

Hai et al (Hai et al., 2023)demonstrated that icariin alleviates nonalcoholic fatty liver disease (NAFLD) in polycystic ovary syndrome (PCOS) models by improving hepatic fatty acid oxidation and inhibiting lipid accumulation. Specifically, icariin upregulated the expression of PPARα and CPT1A, key enzymes in fatty acid metabolism, reducing hepatic steatosis by 64% and providing a novel therapeutic target for metabolic cirrhosis (Hai et al., 2023; Wang et al., 2024; Jin et al., 2025). This finding expands the application scope of icariin to metabolic liver diseases associated with endocrine disorders.

**Figure 6:**
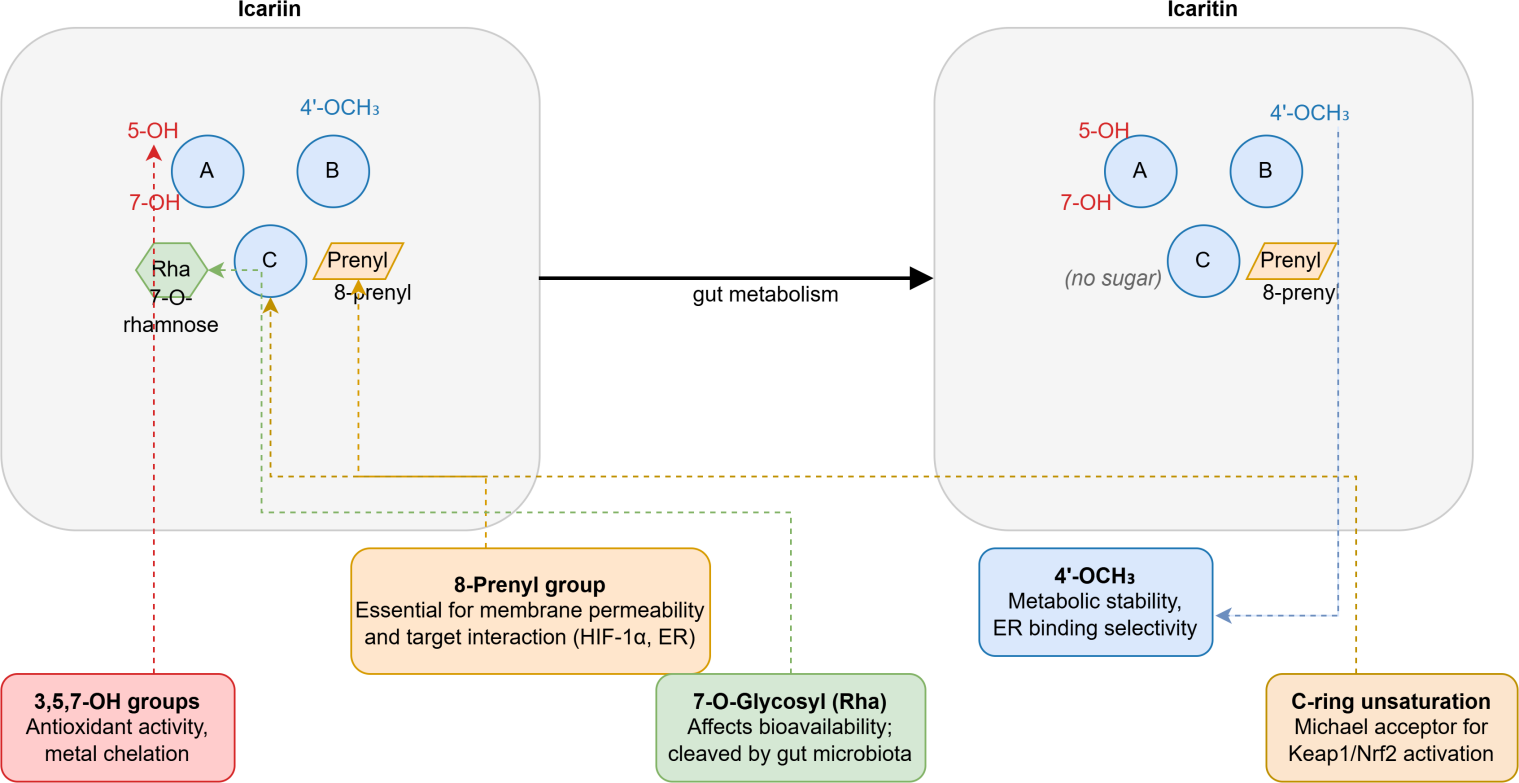
Structure-activity relationship (SAR) of icariin and icaritin. Key difference: glycosylation state.

The absorption and metabolism of icariin and icaritin are critically dependent on gut microbiota. Wu et al (Wu et al., 2016)demonstrated that human intestinal bacteria rapidly metabolize icariin to icariside II and subsequently to icaritin. Icariin itself is minimally absorbed as parent; the active moiety reaching systemic circulation is primarily icaritin and its glucuronidated conjugates (Mou et al., 2024). The chemical structures and metabolic conversion are depicted in **Figure 1**, and the structure-activity relationship is summarized in **Figure 6**.

### 6.2 Hepatotoxicity, dosing uncertainty, and the challenge of translational scaling

Safety concerns emerge at high doses, but the translation of these findings to human cirrhosis is fraught with uncertainty. Wang et al (Wang et al., 2024)demonstrated that high-dose Epimedium extract (>2 g/kg/day in rats) induced cholestatic injury, leading to a tentative recommendation of a 50% dose reduction for Child-Pugh B/C patients. However, this recommendation is based on allometric scaling that does not account for the altered pharmacokinetics inherent to cirrhosis—such as reduced CYP450 metabolism, hypoalbuminemia, and portosystemic shunting—which could lead to either increased toxicity or subtherapeutic exposure.

Similarly, Zhong et al (Zhong et al., 2019) conducted zebrafish toxicity studies and established LC_50_ values of 45–78 μM icaritin, with hepatotoxicity manifesting as steatosis and necrosis at concentrations >30 μM. While these data provide important benchmark toxicity values, their direct translation to human therapeutic dosing is limited by species differences in drug metabolism, bioavailability, and the absence of the cirrhotic microenvironment in these models. The narrow margin between therapeutic and toxic concentrations observed *in vitro* (approximately 2-3-fold) underscores the need for caution, but this should be interpreted as a signal for careful dose titration in future clinical trials, rather than a defined clinical therapeutic window.

A critical knowledge gap, as highlighted in Priority 3 of **Table 3**, is the complete absence of pharmacokinetic data for icaritin in cirrhotic patients. Preliminary data in healthy subjects and animals indicate that after oral administration, free icaritin concentrations are low (Cmax ~1-2 μM in rats), but hepatic accumulation may reach higher levels (~65% of dose distributes to liver) (Sun et al., 2024). Glucuronidated metabolites may serve as circulating reservoirs that undergo deconjugation in inflamed tissues (Szabó et al., 2022; Shen et al., 2024). Until formal pharmacokinetic studies in cirrhotic patients are available—ideally from microdialysis studies or population PK modeling in decompensated cirrhosis—any statements regarding a ‘therapeutic window’ remain hypothetical. The safety and toxicity profile across species, with explicit acknowledgment of translational uncertainties, is provided in **Table 4**.(see at the end of the article).

**Table 3:** Priority matrix for future research on epimedium flavonoids in liver disease.

Research domain	Priority level	Timeline	Key milestones	Evidence gap addressed	Expected impact
HBV/HCV model validation	Critical	2–3 years	Transgenic mouse studies, liver organoids	Tier 3: Absence of viral models	Mechanistic clarity in relevant disease context
Phase III cirrhosis trial	Critical	5–7 years	Paired biopsy RCT, MELD stratification	Tier 3: No cirrhosis trials	Regulatory approval pathway
PK/PD in cirrhosis	High	2–3 years	Microdialysis, PBPK modeling	Tier 3: Undefined PK in cirrhosis	Dose optimization for cirrhotic patients
Drug-drug interactions	High	2–3 years	Formal DDI studies with DAAs	Tier 3: No DDI data	Clinical safety assurance
Biomarker development	Medium	3–4 years	Non-invasive fibrosis assays	Tier 2 → Tier 1 validation	Treatment monitoring tools

**Table 4:** Preclinical and clinical safety signals of epimedium flavonoids: translational uncertainties and knowledge gaps.

Species/model	Toxicity threshold	Hepatotoxic mechanism	Clinical implication
Rat (28-day)	2 g/kg/day (extract)	Cholestatic injury, bile acid accumulation	Provisional allometric scaling; requires validation in cirrhotic patients with altered PK. Reduce 50% for Child-Pugh B/C as a conservative starting point, pending formal PK studies.
Zebrafish (embryo)	LC_50_ 45 μM (icaritin)	Steatosis, necrosis	Benchmark toxicity value for reference; direct human translation limited by species differences and absence of cirrhotic pathophysiology.
Rat primary HSCs	IC_50_ 12.5 μM (selective)	Mitochondrial apoptosis	Therapeutic index suggested in rodents; human relevance uncertain due to interspecies PK/PD differences. Achievable free plasma concentrations in rats are ~1-2 μM, but hepatic accumulation may reach higher levels.
Clinical trials	Grade 3–4 AE 15-25%	Bilirubin elevation (Child-Pugh B)	Observed safety in compensated cirrhosis (Child-Pugh A/B); dose requirements for decompensated cirrhosis (Child-Pugh C) unknown. Monitor liver function tests closely.

## 7 Traditional formulations and real-world evidence

Traditional Chinese medicine formulations containing Epimedium have documented clinical efficacy in specific liver disease subtypes. Chen et al (Chen et al., 2019) conducted a systematic review and meta-analysis protocol to evaluate the efficacy and safety of Yinchen Sini Decoction in treating biliary atresia patients after Kasai portoenterostomy. The study protocol included strict inclusion criteria for randomized controlled trials, aiming to provide high-quality evidence for the application of traditional Chinese medicine in postoperative liver function improvement of biliary atresia patients. This protocol lays a foundation for evidence-based research on TCM formulations in rare liver diseases (Lu et al., 2025).

## 8 Critical knowledge gaps and future research priorities

Despite compelling preclinical mechanistic evidence and promising early-phase clinical signals in HCC, several critical gaps currently preclude the routine clinical application of Epimedium flavonoids in hepatitis and cirrhosis. The following prioritized research agenda addresses these deficiencies:

### 8.1 Priority 1: dedicated viral hepatitis models

Only 2 of 20 reviewed studies employed HBV/HCV-relevant models. The field urgently requires validation specifically in immune-competent, viral-relevant models such as HBV transgenic mice, human liver chimeric models, and HBV-infected primary human hepatocyte cultures to confirm antiviral synergy and fibrosis-specific mechanisms in the absence of a tumor microenvironment, moving beyond the current evidence derived from toxin-induced models and HCC studies (Tang et al., 2023).8.2 Priority 2: Phase III Cirrhosis Trials.

No phase III RCTs specifically target fibrosis/cirrhosis endpoints. A multicenter, randomized, placebo-controlled trial of icaritin in HBV-related compensated cirrhosis (F3-F4) with paired biopsy endpoints is the highest priority. Stratification by Child-Pugh score, MELD, and gut microbiome composition would enable personalized therapy (Xie et al., 2022).

### 8.3 Priority 3: pharmacokinetic characterization in cirrhosis

No data exist on icaritin liver exposure in decompensated cirrhosis. Microdialysis studies in cirrhotic patients and population PK modeling integrating CYP3A4/2C9 polymorphisms and microbiome composition are needed to define therapeutic windows (Zhong et al., 2019; Zhou et al., 2024).

### 8.4 Priority 4: drug-drug interaction assessment

Interaction studies with direct-acting antivirals (e.g., sofosbuvir, velpatasvir) and nucleos(t)ide analogs (e.g., entecavir, tenofovir) are completely absent. Given CYP3A4 involvement, formal interaction studies are mandatory before clinical use in antiviral-treated patients (Luo et al., 2024).

**Figure 7:**
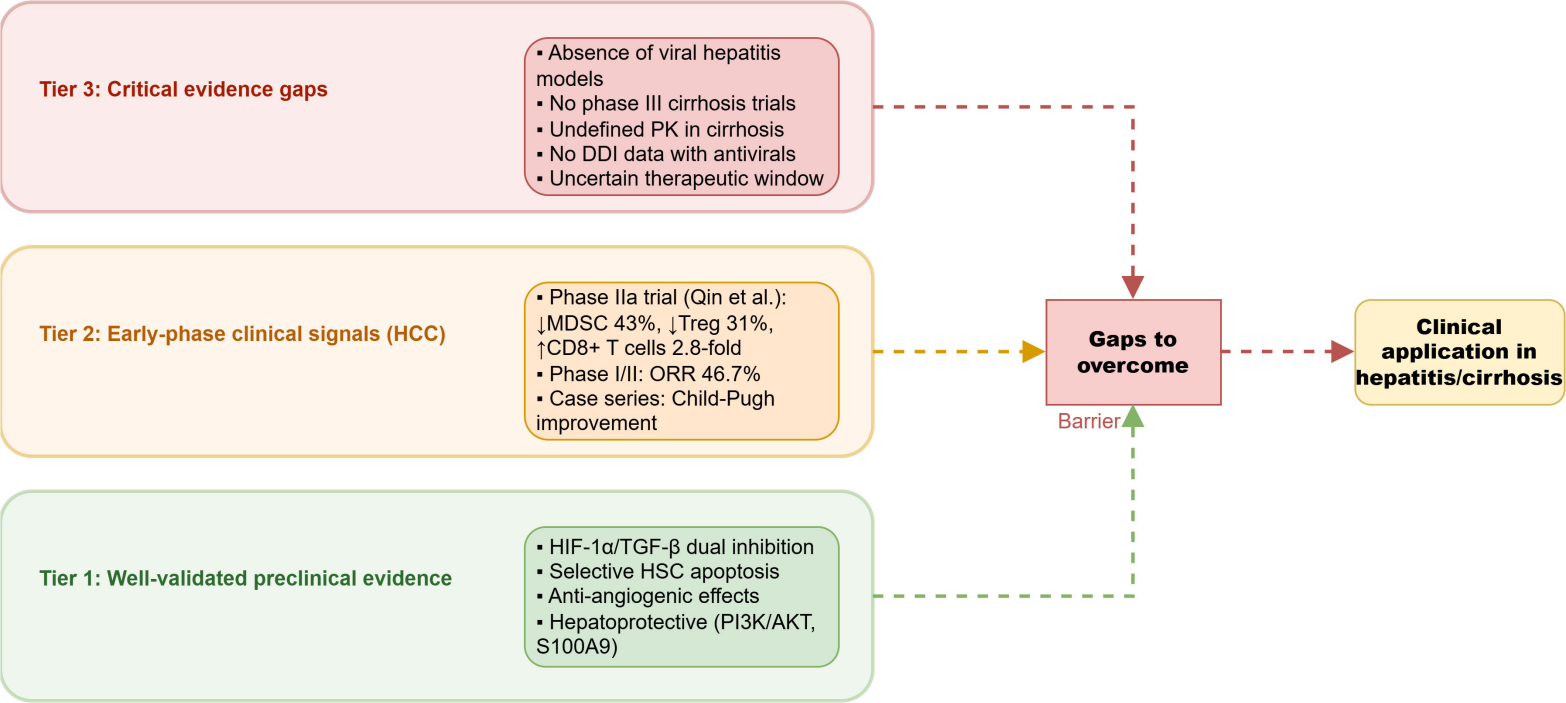
Evidence hierarchy and translational roadmap for Epimedium flavonoids in liver disease Research priorities to bridge gaps: 1. HBV/HCV model validation (2–3 yrs) 2. Phase III cirrhosis trial (5–7 yrs); 3. PK/PD in cirrhosis (2–3 yrs); 4. Drug-drug interaction studies (2–3 yrs); * Tier 2 data derived from HBV-related HCC trials; not directly translatable to chronic hepatitis/cirrhosis.

A priority matrix for future research on Epimedium flavonoids in liver disease is provided in **Table 3** (see at the end of the article), outlining key domains, timelines, and expected impacts. The integrated molecular mechanisms and translational roadmap are visually summarized in **Figure 7** and **3**.

## 9 Discussion

### 9.1 Strengths of current evidence base

The reviewed literature demonstrates remarkable consistency in preclinical mechanistic findings across multiple independent laboratories and disease models. The convergence on TGF-β/Smad, Nrf2/HO-1, and HIF-1α pathways, validated by genetic knockout and rescue experiments, provides high confidence in these molecular targets (Lu et al., 2023). The demonstration of selective HSC apoptosis (Kim et al., 2017) and anti-angiogenic effects (Fan et al., 2019) offers a compelling therapeutic window. Clinically, the immunomodulatory effects in HBV-related HCC (Bi et al., 2022) and the direct cirrhosis modification suggested by Child-Pugh improvements (Xie et al., 2022) are particularly encouraging.

### 9.2 Limitations, evidence gaps, and barriers to clinical translation

To provide clarity on the current state of knowledge, we have classified the evidence into three tiers:

**Tier 1: Well-Validated Preclinical Mechanistic Evidence**.

- Anti-fibrotic mechanisms: HIF-1α/TGF-β dual inhibition, selective HSC apoptosis, anti-angiogenic effects. These findings are supported by multiple independent laboratories, genetic knockout/rescue experiments, and across several toxin-induced and metabolic models (Qin et al., 2020).
- Hepatoprotective mechanisms: PI3K/AKT/Bcl-2 anti-apoptotic signaling, S100A9/TLR4 blockade. These mechanisms are well-validated in acute injury models (Tang et al., 2023).

**Tier 2: Early-Phase Clinical Signals (Hypothesis-Generating).**

- HBV-related HCC: Immunomodulatory effects (MDSC/Treg reduction, CD8+ T-cell enhancement) observed in a Phase IIa trial (n=45) and supported by case series (n=23, n=15). Objective response rates of 46.7% in a Phase I/II trial These data provide proof-of-concept but are limited by small sample sizes, absence of control arms, and the oncological context, which is not directly translatable to chronic hepatitis or cirrhosis (Szabó et al., 2022).
- Child-Pugh improvement: Observed in a small case series (Bi et al., 2022), but requires confirmation in controlled trials with paired biopsy endpoints.

**Tier 3: Critical Evidence Gaps Preventing Routine Clinical Application.**

- Absence of viral hepatitis models: Only 2 of 33 reviewed studies employed HBV/HCV-relevant models.
- No phase III cirrhosis trials: No RCTs with fibrosis/cirrhosis as the primary endpoint.
- Undefined pharmacokinetics in cirrhosis: No data on icaritin exposure in decompensated cirrhosis.
- No drug-drug interaction studies: Complete absence of data with direct-acting antivirals or nucleos(t)ide analogs.
- Uncertain therapeutic window: Translational uncertainty from *in vitro* and animal data to human cirrhosis; achievable free plasma concentrations (1-2 μM in rats) are lower than many *in vitro* IC_50_ values (5-20 μM), though hepatic accumulation and conjugated metabolites may partially bridge this gap.

This hierarchical presentation clarifies that while the mechanistic foundation is well-supported, the clinical evidence remains preliminary, and substantial gaps must be addressed before Epimedium flavonoids can be considered for routine clinical use in hepatitis and cirrhosis.

### 9.3 Therapeutic potential versus clinical reality

The pleiotropic “multi-target, weak-binding” paradigm offers distinct advantages over single-target biologics: reduced resistance potential and favorable safety profiles at therapeutic doses. However, this mechanistic complexity complicates regulatory approval pathways and dose optimization strategies. Unlike monoclonal antibodies with defined targets, the pharmacodynamic biomarkers for icaritin remain poorly characterized, hindering precise dose selection (Fan et al., 2019).

Cost-effectiveness considerations also remain unaddressed. While softgel formulations improve bioavailability, manufacturing complexity and the potential need for microbiome-guided dosing may increase costs compared to conventional small molecules. Formal health economic analyses will be essential for widespread adoption in resource-constrained healthcare systems (Bi et al., 2022).

### 9.4 Integration with contemporary standard of care

Epimedium flavonoids should not be conceptualized as replacements for antiviral therapy but as complementary agents addressing residual fibrosis risk despite viral suppression. Potential integration strategies include: add-on therapy to entecavir/tenofovir in HBV patients with advanced fibrosis (F3-F4); sequential therapy following viral cure to promote fibrosis regression; and combination therapy with statins or other anti-fibrotics in NASH-related cirrhosis. The favorable oral administration route and established safety profile position icaritin advantageously for long-term cirrhosis management, unlike injectable immunotherapies that pose adherence challenges (Bi et al., 2022).

## 10 Conclusions

This integrative review demonstrates that Epimedium flavonoids, particularly icaritin, possess a compelling multi-target therapeutic profile for hepatitis and cirrhosis, underpinned by well-validated mechanistic evidence of anti-fibrotic, hepatoprotective, and immunomodulatory mechanisms. Early-phase clinical signals in HBV-related HCC provide proof-of-concept for immune modulation and have advanced clinical translation in the oncological setting. However, critical evidence gaps—most notably the absence of validation in viral hepatitis models, the lack of phase III cirrhosis trials, undefined pharmacokinetics in cirrhotic patients, and the complete absence of drug-drug interaction data with standard-of-care antivirals—currently prevent routine clinical application in hepatitis and cirrhosis.

The convergence of traditional botanical medicine experience, modern mechanistic elucidation, and emerging clinical data creates a robust rationale for advancement. If the prioritized research agenda outlined in **Table 3** is executed with scientific rigor—addressing the evidence gaps identified above—Epimedium flavonoids could emerge as a potential new class of orally active, plant-derived anti-fibrotic agents. At present, however, their use in hepatitis and cirrhosis remains strictly investigational. The path forward demands rigorous, large-scale clinical investigation that respects both the complexity of cirrhosis pathophysiology and the pleiotropic nature of these promising botanical compounds.

## Glossary

ALT: alanine aminotransferase
APAP: acetaminophen
BID: twice daily
CCl₄: carbon tetrachloride
cGAS: cyclic GMP-AMP synthase
CTCAE: Common Terminology Criteria for Adverse Events
ERα: estrogen receptor-α
FAO: fatty acid oxidation
GPX4: glutathione peroxidase 4
GSDMD: gasdermin D
HCC: hepatocellular carcinoma
HIF-1α: hypoxia-inducible factor-1α
HSC: hepatic stellate cell
IFN: interferon
IL: interleukin
IRF3: interferon regulatory factor 3
iNOS: inducible nitric oxide synthase
L-FABP: liver fatty acid–binding protein
LC₃: microtubule-associated protein 1 light chain 3
MELD: Model for End-Stage Liver Disease
MDSC: myeloid-derived suppressor.
Model: Key Molecular TargetsCCl₄-induced (rat), HIF-1α, TGF-β/Smad, Bax/Bak-1
TAA-induced (rat): VEGF/VEGFR2, LC3-II/p62
NAFLD (PCOS rat): PPARα, CPT1A, FAO enzymes
